# Drug Delivery Approaches for the Treatment of Cervical Cancer

**DOI:** 10.3390/pharmaceutics8030023

**Published:** 2016-07-20

**Authors:** Farideh Ordikhani, Mustafa Erdem Arslan, Raymundo Marcelo, Ilyas Sahin, Perry Grigsby, Julie K. Schwarz, Abdel Kareem Azab

**Affiliations:** 1 Department of Radiation Oncology, Cancer Biology Division, Washington University School of Medicine, Saint Louis, MO 63108, USA; Faride.ordikhani@gmail.com (F.O.); marslan@radonc.wustl.edu (M.E.A.); raymundo.marcelo@wustl.edu (R.M.); jschwarz@radonc.wustl.edu (J.K.S.); aazab@radonc.wustl.edu (A.K.A.); 2 Department of Medicine, Mount Auburn Hospital, Harvard Medical School, Cambridge, MA 02138, USA; ilyassahin@gmail.com; 3 Department of Radiation Oncology, Radiology and Obstetrics and Gynecology, Washington University School of Medicine, Saint Louis, MO 63108, USA; pgrigsby@radonc.wustl.edu; 4 Alvin J. Siteman Cancer Center, Washington University School of Medicine, Saint Louis, MO 63108, USA; 5 Department of Radiation Oncology, Cell Biology and Physiology, Washington University School of Medicine, Saint Louis, MO 63108, USA

**Keywords:** cervical cancer, drug delivery systems, systemic delivery, local delivery, chemotherapy

## Abstract

Cervical cancer is a highly prevalent cancer that affects women around the world. With the availability of new technologies, researchers have increased their efforts to develop new drug delivery systems in cervical cancer chemotherapy. In this review, we summarized some of the recent research in systematic and localized drug delivery systems and compared the advantages and disadvantages of these methods.

## 1. Introduction

Cervical cancer is the third most common malignancy among women, with approximately half a million newly diagnosed cases and over 200,000 deaths annually [1,2]. Although most cases of cervical cancer can be prevented by routine screening and treatment of precancerous lesions, cervical cancer is the leading cause of cancer mortality among women in developing countries [3]. Its high number of deaths is attributable to the extremely low survival rates of patients with advanced cervical cancer at diagnosis [2].

The treatment of cervical cancer is stage-specific. While early stage disease can be cured with radiotherapy or surgery, the most effective treatment for locally advanced stage patients is concurrent chemotherapy and pelvic irradiation [4,5]. Typically, once weekly cisplatin is administered intravenously while a combination of external beam radiation and brachytherapy is used to treat the pelvic tumor. Recent findings from large scale genomic sequencing of human cervical tumors has suggested that targeted therapies may present a better option [6]. Of note, it was recently shown that addition of angiogenesis inhibitor, bevacizumab, to combination chemotherapy in patients with recurrent, persistent, or metastatic cervical cancer improved median overall survival [7].

**Figure 1:**
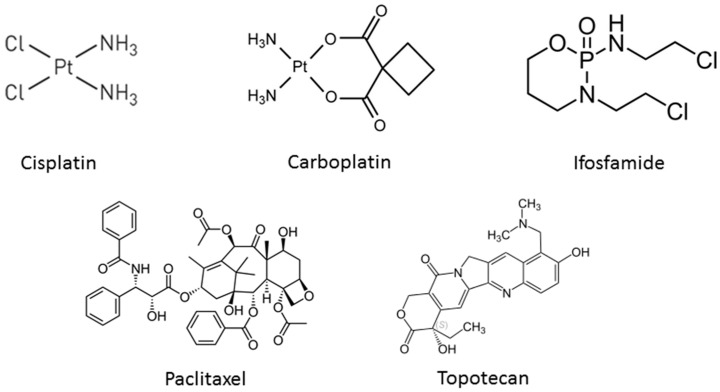
The chemical structures of commonly used chemotherapeutic drugs in cervical cancer.

Despites advances in treatment, patients with metastatic cancers and those with recurrent or persistent disease have limited treatment options [8]. A number of chemotherapeutic agents have shown activity in advanced and metastatic cervical cancer, including cisplatin [9], carboplatin [10], paclitaxel [11], ifosfamide [12], and topotecan [13]. The chemical structures of these drugs are shown in Figure 1. Among these chemotherapeutic agents, cisplatin is considered the most effective chemotherapeutic drug for advanced cervical cancer [4]. Its efficacy is due to its induction of oxidative stress and apoptosis in tumor cells through its direct interaction with DNA forming adducts, which inhibit gene transcription [14]. However, its clinical use is limited due to tumor resistance and serious side effects like thrombocytopenia, neutropenia, nephrotoxicity, neurotoxicity, anemia due to hematological toxicity, and bone marrow depression [15,16]. 

Drug delivery approaches can be categorized based on their route of administration (systemic or localized) and the type of device. Systemic delivery is based on particles (dendrimer, micelle, liposome, nano/microparticle) with surface features that help target the desired site when injected. In contrast, localized delivery limits systemic drug toxicities by direct delivery of the drug to the tumor. Based on depot systems that are implanted either directly into or adjacent to the tumor, the later promotes the release of drug directly to the cancer site [17,18]. In recent years, numerous studies have been done with localized drug delivery strategies to treat cervical cancer. Although these strategies could reduce systemic toxicity, significant improvement in delivery strategies is still necessary to increase patient compliance and reduce chemotherapy-related side effects. Fortunately, the easily-accessible cervix permits non-invasive implantation directly into the cancerous tissue at the time of brachytherapy implant [19].

In this review, we discuss the literature supporting novel drug delivery strategies for cervical cancer treatment and highlight some of the current advancements in systemic and local drug delivery systems.

## 2. Systemic Drug Delivery Systems

**Figure 2:**
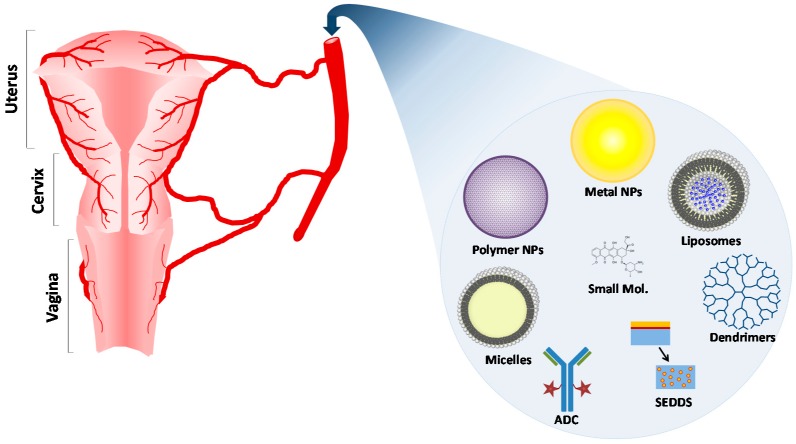
Systemic Drug Delivery systems used in cervical cancer, including metal nanoparticles, polymer nanoparticles, liposomes, micelles, Antibody–Drug Conjugates (ADCs), Self-Emulsifying Drug Delivery Systems (SEDDSs) and dendrimers.

The entrapment of chemotherapeutic drug in nanocarriers has attracted considerable attention due to their structure, varied composition, and surface modifications [20]. The most common architectures for targeted drug delivery applications are nanoparticles, liposomes, micelles, and dendrimers (Figure 2). The size of these particles usually ranges from 10 to 150 nm, which ensures increased accumulation in the tumor with longer circulation time. Particle sizes of less than 10 nm would be rapidly cleared by the kidneys, and sizes larger than 150 nm would risk recognition and elimination by the macrophage cells [20]. Encapsulating chemotherapies in nanocarriers offers a number of advantages, such as protection from degradation in the bloodstream, enhancement of drug stability, targeted drug delivery, decreased toxic side effects, and improved bioavailability of the drug [21,22]. Nanoscale drug delivery systems have been shown to enhance drug specificity, decrease systemic drug toxicity, improve absorption rates, and provide protection for active agents from biological and chemical degradation [23,24]. Moreover, they can be designed as controlled and/or sustained drug release systems to deliver therapeutics at a predetermined rate, for a specified period of time. Controlled drug delivery systems could increase patient compliance by reducing the need for repeated drug administration [25]. Little is known about the effective concentrations of drugs used with the cervix tissue, however, it is suggested that controlled released formulations with longer release will increase the local concentration of the drugs and increase efficacy of the chemotherapies.

Human cancer cell lines are fundamental models, either in vitro as monolayer culture or in vivo as xenografts in mice, to study the efficacy of therapeutic agents in cancer therapy. Hela was the first cultured cancer cell line, which was derived from cervical cancer cells taken from Henrietta Lacks in 1951 [26]. Since then, several cervical cancer cell lines including SiHa, CaSki, C-33A, and ME-180 were established [27]. It is worth to mention that these cell lines do not have equal value as tumor models, thus the in vitro results of drug efficacy experiments are different. On the other hand, cancer cell lines in three-dimensional (3D) culture models indicate higher malignancy, invasive characteristic, and drug resistance than classic two-dimensional (2D) cell culture [28]. Thus, limited success at translating new drug delivery systems to the clinics could be related to limitations of classic 2D cell culture and drug screening models [29].

### 2.1. Inorganic Nanocarriers

Nanoparticles (NPs) of noble metals have proven their efficacy in the clinical field for cancer therapy due to their unique features such as ease of synthesis, simple surface chemistry and functionalization, broad optical properties, and high surface-to-volume ratio [30]. Noble metal NPs are versatile agents with a variety of biomedical applications, including biodiagnostics [31], imaging [32], photothermal therapy [33], radiotherapy enhancement [34], and gene and drug delivery [35,36]. Recently, the antitumor potency of NPs against some cell lines has been reported [37,38,39]. Their effectiveness comes from their interaction with cells, where cellular uptake leads to oxidative stress from the generation of reactive oxygen species, as well as their interaction with intracellular macromolecules like DNA and proteins, since NPs can easily traverse the nuclear membrane and directly or indirectly interact with DNA, though by a still unknown mechanism [37].

Noble metal NPs are mostly made by physical and chemical methods; however, this expensive synthesis can allow toxic substances to be absorbed onto their surfaces [40]. This problem is overcome by alternative biological synthesis. The green synthesis of NPs is safe, cost-effective, and eco-friendly [41], without needing elaborate processes like multiple purification steps and microbial culture maintenance [37].

One of the most promising biomaterials in the field of nanomedicine is silver nanoparticles (AgNPs) [40,42]; its antitumor potency against cervical cancer cell lines has been recently explored. Biogenic AgNPs decreased cell proliferation and increased DNA damage, intracellular reactive oxygen species, and apoptosis, leading to cancer cell death [41]. Other studies demonstrated that particle size, dose, and time influence their toxicity [37], and that, against HeLa cervical cancer cells, AgNPs loaded with *Moringa olifea* extract showed anticancer activity [43].

Unfortunately, silver was shown to be reactive with body tissues, therefore, as a strategy to reduce the high reactivity and adverse effects of silver, AgNPs were encapsulated in gelatin or polyethylene glycol to increase the biocompatibility of the surface. However, these coated particles were less effective than the non-coated ones [44]. In another experiment, silver-core and protein-lipid-shell NPs exhibited anti-proliferative activity against HeLa cells due to enhanced cell penetration and targeted action [45].

Gold (Au) NPs emerged as a promising scaffold for chemotherapeutic drug and gene delivery vehicle, due to its high bioavailability and low immunogenicity [46]. AuNPs loaded with *Podophyllum hexandrum* exhibited an effective in vitro anti-proliferative activity against HeLa cells by induction of DNA damage and cell cycle arrest at G2/M. Further results demonstrated that the mitochondria of AuNPs-treated cells became dysfunctional due to the activation of the caspase cascade, leading to apoptosis [47]. When conjugated to gallic acid, AuNPs exhibited cytotoxicity in both Human Papilloma Virus (HPV) negative C33A cervical cancer cells and HPV type 16-positive (CaSki) or HPV type 18-positive (HeLa) cervical cancer cells, but not in normal cells in vitro [48]. Moreover, Au NPs loaded with doxorubicin exhibited stronger anticancer activity on human cervical cancer cell lines compared to free drug [49].

To improve the selective delivery of therapeutic agents to specific cells or tissues, targeting ligands (i.e., antibodies [50], aptamers [51], peptides [52,53], or small molecules [54]) are attached to the surface of the nanocarriers; which allows preferential accumulation of the nanocarriers in specific cells or tissues [55,56]. Au NPs derivatized with rhetinoic acid showed to improve the dug potency and cell growth inhibition up to 6 times compared to non-targetd Au NPs [57].

Other examples of biomaterials are tea polyphenol-functionalized platinum NPs (TPP@Pt), which inhibited the proliferation of and induced chromatin condensation and nuclear fragmentation of SiHa cells [58], and copper(II) complex (LQM402), which exhibited a cytotoxic effect against cell lines and selectivity for HeLa and CaSki cells, while displaying less cytotoxicity against normal fibroblasts [59].

### 2.2. Polymeric Nanoparticles 

Biodegradable polymeric NPs have received considerable research interest in anticancer drug delivery due to their high drug loading capacity, self-stability, high cellular uptake, more desirable biodistribution, and capability to deliver both hydrophilic and hydrophobic drugs [60,61]. While the stealth polymers surrounding these NPs prolong circulation time, their dense layer of polymers could inhibit the ability of target cancer cells to uptake anticancer drugs [24].

Biodegradable polymers, either natural or synthetic, can break down through chemical or enzyme-catalyzed degradation. Biodegradable polymers offer numerous advantages in the field of drug delivery: (1) The drug release kinetics can be controlled by degradation rate of polymers, so a sustained and controlled drug release is possible; (2) the polymeric carrier would degrade into nontoxic, absorbable subunits that can be metabolized; and (3) there is no need for a follow-up surgical removal once the drug supply is depleted [62].

Nanoparticles of various polymers have been tested. One example made of different derivatives of poly(lactide-*co*-glycolide) (PLGA) showed sustained and controlled delivery of docetaxel for cervical cancer treatment both in vitro and in vivo and demonstrated higher cellular uptake efficiency and high antitumor efficacy [23,61,63,64,65]. Similarly, the acrylic polymers Eudragit-E and polyvinyl alcohol (PVA) loaded with Naringenin induced changes in mitochondrial membrane potential, augmented reactive oxygen species levels, decreased intracellular glutathione levels, produced morphological alterations in apoptosis, and caused dose-dependent cytotoxicity [66]. In another study, genistein-encapsulated ε-caprolactone-based NPs exhibited more cytotoxicity and tumor cell growth inhibition compared with pristine genistein in the subcutaneous HeLa xenograft tumor model in BALB/c nude mice [67].

A potential therapeutic target in cervical cancer is the folate receptor given its overexpression in human cervical cancer cells [60,68]. NPs that were conjugated with folic acid to l-tyrosine-polyphosphate [69], gelatin [60], chitosan [70], or chitosan-coated PLGA nanoparticles [71] and loaded with silver carbene complex, cisplatin, selenocystine, or carboplatin, respectively, increased the specificity of chemotherapeutic drugs up to 10-fold greater than control NPs without drug in cervical cancer cells. In vivo antitumor activity results of folate-targeted doxorubicin-loaded NPs exhibited improved targeting and anti-tumor efficacy in inhibiting tumor cells [68]. In a recent study, pullulan acetate NPs decorated with folate were used as a carrier for treating cervical carcinoma and its metastatic hepatocellular carcinoma [72].

### 2.3. Micelles

Made up of amphiphilic block copolymers, polymeric micelles are colloidal particles that can assemble themselves [73]. They are important for cancer therapeutic applications due to their in vivo stability, ability to solubilize water-insoluble drugs, prolongation of blood circulation time, and small size of 10 to 100 nm [74,75]. For example, polymeric composite micelles, which were targeted with folic acid and loaded with paclitaxel, inhibited tumor growth and caused cell apoptosis of U14 cervical cancer tumors both in vitro and in vivo [76].

Polymeric micelle of candesartan-*g*-polyethyleneimine-*cis*-1,2-cyclohexanedicarboxylic anhydride polymer loaded with paclitaxel has negative surface charges and a diameter of about 100 nm; showed strong antitumor efficacy by mediating amidase-responsive drug-release manners and quick endosomal escape [77].

### 2.4. Liposomes

One of the most studied nano-carriers is liposomes [78], which are single lipid bilayer vesicles that encapsulate water-soluble drugs in an aqueous core while the lipidbilayer entangles lipid-soluble drugs [74].

Liposomes were used to deliver anticancer agents including bleomycin sulfate [79], cisplatin [80], and curcumin to treat cervical cancer and could enhance bioavailability, stability, and cancerous cellular uptake of encapsulated chemotherapeutic agents [81].

Liposomal cisplatin exhibited potent antitumor activity on ME-180, R-ME-180 (ME-180 cisplatin-resistant clone), and HeLa cells. Compared to free cisplatin, liposomal cisplatin actively inhibited cell proliferation and decreased the spheroid-forming ability of R-ME-180 cells in tumors and in nude mice, in a dose-dependent manner [80].

Transferrin-targeted liposomes where shown to be more specific in delivery of paclitaxel to cervical cancer cell lines, compared to non-targeted liposomes [82]. Moreover, in vitro studies in human cervical carcinoma cell line (HeLa) showed liposomes targeted with folic acid and transferrin had higher cell association, penetration and efficacy of delivering doxorubicin compared to either of the single-ligand targeted liposomes, or non-targeted liposomes [83].

### 2.5. Dendrimers

Dendrimers are spherical, highly symmetric, and greatly branched, macromolecules with a well-defined structure, surface charge, and molecular size that display a high degree of monodispersity [84,85]. Their structure permits the attachment and presentation of antigen molecules at their periphery, causing them to be exceedingly multifunctional. Drugs can be loaded into cavities in their cores by chemical linkages, hydrophobic interactions, hydrogen bonds, or conjugation to the polymer scaffold [86]. For example, they can be used to overcome the poor immunogenicity of peptide-based vaccines against cervical cancer as shown by a study that developed a polyacrylate star-polymer conjugated to HPV E7 protein. It was shown that these conjugates alone and after a single immunization were able to diminish tumor growth and eliminate E7-expressing TC-1 tumors in mice [87].

Doxorubicin loaded dendrimer was conjugated with two of cancer cell targeting moieties, IL-6 antibody and RGD (Arginyl-glycyl-aspartic acid) peptide and the drug loading capacity and release profile as well as their targeting efficiency were compared. Drug loaded dendrimers decorated with IL-6 antibody exhibited higher cellular internalization, lower IC_50_ value, higher drug loading, faster drug release rate and more cytotoxicity compared to RGD-conjugated one in HeLa cells. This could be probably because of the higher multivalent ligand density on the surface of the IL6-conjugated dendrimers, which cause better drug delivery through receptor-mediated endocytosis [88].

### 2.6. Self-Emulsifying Drug Delivery Systems

Another approach to improve the delivery of highly lipophilic drugs is by self-emulsifying drug delivery systems (SEDDS) [89]. SEDDS are complex formulations consisting of oil, surfactant, co-surfactant, cosolvent, and drug; upon contact with aqueous medium these isotropic preconcentrates spontaneously generate coarse emulsions, or fine nano-emulsions, referred to as self-nanoemulsifying drug delivery systems (SNEDDS) [90]. SEDDS offers numerous advantages including quick onset of action, enhancing bioavailability, minimizing side effect, control of delivery profiles, ease of manufacturing, and protection of sensitive therapeutics such as peptides, which are prone to enzymatic hydrolysis [91,92,93]. A few studies have shown the application of SEDDS in cervical cancer treatment; SEDDS containing antitumor agents (bleomycin, cisplatin and ifosfamide) exhibited an increase in the inhibitory effect of the drugs in a concentration-dependent manner on Hela cells [94]. In another study, SEDDS formulation was used to enhance water-solubility and bioavailability of curcumin. Oral bioavailability studies in male Wistar rats exhibited 26-fold more absorption of curcumin via its delivery through SEDDS [95].

### 2.7. Antibody–Drug Conjugates

Targeted delivery of drugs by conjugating them to monoclonal antibodies, which can bind specifically to tumor-associated target antigens is an innovative approach in cancer treatment that gains considerable research interest. Antibody–drug conjugates (ADCs) can selectively deliver therapeutics to tumor cells and provide sustained clinical benefit to cancer patients with less systemic toxicity [96]. Furthermore, ADCs allow more delivery of the drug over repeated cycles of therapy, and consequently improving the therapeutic index, or ratio of efficacy to toxicity of the drug [97]. Although ADCs approaches are promising, a few of them are approved for clinical use such as Brentuximab vedotin (Adcetris^®^) and ado-trastuzumab emtansine (Kadcyla^®^) [98,99]. Insufficient understanding of ADCs mechanism of action, inadequate knowledge of the management and understanding of ADCs off-target toxicities, and difficulties in the selection of suitable clinical settings such as patient selection, dosing regimen are some possible explanations for the slow clinical translation of new ADCs [97]. TF-011-MMAE (HuMax-TF-ADC), an antibody drug conjugate targeting tissue factor-specific cells, exhibited excellent antitumor activity in patients with advanced solid cancers, including cervical cancer [100]. Another new clinical trial of ADCs is IMMU-132, which is targeting the TROP-2 antigen and expressed by many human solid tumors including breast, colon and rectum, lung, pancreas, ovary, prostate, and cervical cancers [101,102].

## 3. Localized Drug Delivery Systems

**Figure 3:**
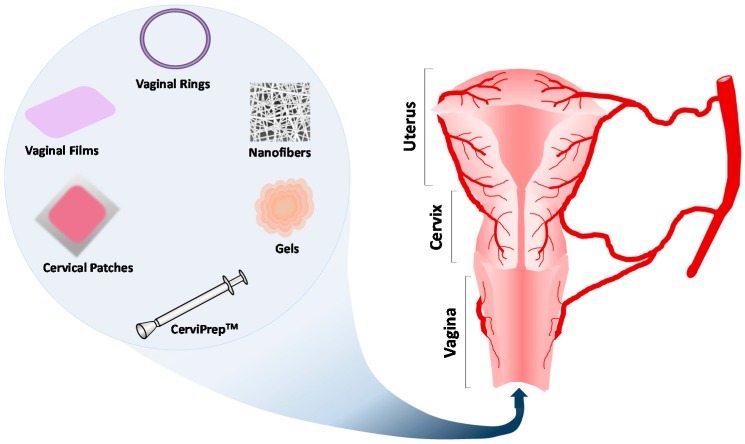
Drug Localized Drug Delivery systems used in cervical cancer, including vaginal rings, vaginal films, cervical patches, nanofibers, gels and CerviPrep™.

Localized delivery of chemotherapeutic drugs to the cervix offers a number of advantages compared to systemic delivery such as its avoidance of systemic chemotherapeutic drug circulation, which leads to less drug waste and decreased side effects and delivery of high dose of the active agent in the cervix which improves the efficacy of the treatment [18] (Figure 3). The use of local drug delivery systems for the treatment of metastatic cervical tumors may be inefficient, where the disease is disseminated in distant organs, which implies the need of a more systemic approach. However, recent reports show that less than 20% of the cases appear with distant metastasis [103], which emphasizes that most of the cervical cancer cases would benefit from localized drug delivery systems.

This direct delivery is possible due to the easily reachable location of the cervix through the vagina [104]. Presently, different drugs in various forms such as gels, rings, fibers, and tablets are delivered through the vagina for purposes such as contraception or the treatment of fungal, bacterial, and sexually transmitted infections [105,106,107]. Many of these methods have already been studied for use in chemotherapeutic localized delivery, and, with such a wide range of formulations, patient treatment can be individualized by type and dosing of the drug regimen. Improving the overall quality of patients’ lives, localized delivery helps patients recover more quickly and reduces the number of hospital presentations and admissions, decreasing global healthcare system costs [19].

### 3.1. Intra-Vaginal Rings 

As flexible and torus-shaped delivery systems, intra-vaginal rings can provide both sustained and controlled drug release, lasting for several weeks to several months [108]. Recently, intra-vaginal rings have been used for the localized delivery of a chemotherapeutic drug to the cervix with the potential to reduce the need for surgical intervention. Poly(ethylene-*co*-vinyl acetate) ring device incorporated with cisplatin for local treatment of cervical cancer were recently reported. The intra-vaginal rings were found to be effective against both HPV positive and HPV negative cervical cancer cells in vitro [104]. Other thermoplastic vaginal rings were shown to hold and release disulfiram, an anticancer drug, at levels far beyond the IC_50_ value for HeLa cells [108].

### 3.2. Nanofibers

Nanofibers have various applications, one of which is drug delivery, especially in local chemotherapy. Their attractive characteristics for electrospinning used in drug delivery are high encapsulation efficiency, ease of operation, cost-effectiveness, high loading capacity, and simultaneous delivery of various therapies [109]. Recently, drug-loaded ultrafine fibers have been used in local chemotherapy of cervical cancers. Biodegradable polylactide fiber mats loaded with paclitaxel showed strong inhibition of xenograft U14 cervical cancer [110]. At the same drug level, the in vivo trials of cisplatin-loaded biodegradable poly(ethylene oxide)/polylactide composite electrospun nanofibers demonstrated more antitumor efficacy with better systemic safety than the IV injection group [2], indicating the benefits of localized delivery over systematic delivery.

### 3.3. Vaginal Films

Vaginal films made are promising delivery systems that could achieve better patient compliance and therapeutic efficacy. Curcumin-hydroxypropyl cyclodextrin complex vaginal films were shown to be retained in the vaginal mucosa for up to six hours, making it a potentially effective therapy for HPV-induced cervical cancer [111].

### 3.4. Gels

As proven and accepted therapeutics, vaginally-applied gels have drugs and active ingredients which can restore physiological pH, moisturize and lubricate, be a contraceptive or labor inducer, and/or have microbicide activity [106]. In regards to treatment of HPV-induced cervical cancer, a vaginal gel using the biodegradable thermosensitive polymer Pluronic^®^ F127 combined with 5-fluorouracil and alternative mucoadhesive polymers like Carbopol 934, hydroxypropylmethylcellulose, and hyaluronic acid was created. Cytotoxicity studies with HeLa cells showed that complexes of 1% 5-fluorouracil and b-cyclodextrin or hydroxypropyl-b-cyclodextrin were just as effective as free 5-fluorouracil. These results indicate that a lower dose of this anticancer drug can achieve better therapeutic efficacy and increased patient compliance [112]. Although gels are tolerated more than other dosage forms, they can easily leak out of the vagina [2].

### 3.5. Cervical Patches

Another approach to the cervical delivery of a cytotoxic agent is using bioadhesive cervical patches [113]. In one study, a patch containing 20 mg of 5-fluorouracil was paired with cervical tissue samples for a 24-hour period. After this exposure, the tissue concentration of 5-fluorouracil was found to be 100 times that of the determined cytotoxic drug concentration. This outcome shows that for areas of the cervical stroma where pre-cancerous lesions can occur, the patch delivery system could provide clinically effective drug concentrations [114]. However, the turnover of the mucosal lining limits these patches [104].

### 3.6. CerviPrep™

Hodge et al. developed a new cervical delivery instrument (CerviPrep™) that consisted of a tubular applicator with a cervical cap on one end. When the cervical cap was positioned over the patient’s cervix, this device coulddirectly apply pharmaceuticals to the cervix with limited exposure to and absorption by the adjacent vaginal tissue. The results of clinical studies with the CerviPrep™ delivery device showed limited systemic exposure and no toxicities [115].

## 4. Conclusion and Future Direction

Inorganic, lipidic and polymeric nanocarriers are promising candidates for the development of systemic delivery systems in cervical cancer chemotherapy. Experimental results show great potential for the widespread adoption of nanocarriers in cervical cancer treatment over conventional chemotherapy. Their attractive properties include biocompatibility, low toxicity, lower clearance rates, the ability to target specific tissues, and controlled release of chemotherapeutic agents. However, the toxicology of nanocarriers in humans still needs to be fully studied. On the other hand, localized delivery of chemotherapeutic drugs to the cervix offers a number of advantages such as increased efficacy and decreased side effects due to direct delivery to the site of cancer, which avoids systemic circulation of chemotherapeutic drugs. However, the local drug delivery systems are not effective in metastatic tumors. The therapeutic use of these drug delivery devices and formulations would vary depending on the patient’s cancer stage. Any vaginal drug delivery device or formulations could be used to provide local delivery of a chemotherapeutic drug to the cervix, and each has its own advantages like improved patient compliance and disadvantages like expensive production. Although, localized delivery of chemotherapeutic drugs directly to the cervix will improve the patient’s overall quality of life and reduce hospital presentations and cost, more research needs to be performed characterizing the local accumulation and efficacy of drugs in the cervix.
